# Use of Impella Devices for Acute Cardiogenic Shock in the Perioperative Period of Cardiac Surgery

**DOI:** 10.21470/1678-9741-2021-0398

**Published:** 2023

**Authors:** Serge Sicouri, Vishal N. Shah, Meghan Buckley, Nicholas Imperato, Jacqueline McGee, Elena Casanova, Eric Gnall, Konstadinos A. Plestis

**Affiliations:** 1 Lankenau Institute for Medical Research, Wynnewood, PA, USA.; 2 Department of Cardiothoracic Surgery, University of Nebraska Medical Center, Omaha, Nebraska.; 3 Department of Cardiothoracic Surgery, Thomas Jefferson University Hospital, Philadelphia, Pennsylvania.; 4 Division of Cardiology, Lankenau Medical Center, Wynnewood, PA, USA.

**Keywords:** Extracorporeal Membrane Oxygenation, Shock, Cardiogenic, Heart Ventricles, Aortic Valve, Hemodynamics, Cardiac Surgical Procedures, Perioperative Period

## Abstract

**Introduction:**

The Impella ventricular support system is a device that can be inserted percutaneously or directly across the aortic valve to unload the left ventricle. The purpose of this study is to determine the role of Impella devices in patients with acute cardiogenic shock in the perioperative period of cardiac surgery.

**Methods:**

A retrospective single-surgeon review of 11 consecutive patients who underwent placement of Impella devices in the perioperative period of cardiac surgery was performed. Patient records were evaluated for demographics, indications for placement, and postoperative outcomes.

**Results:**

Impella devices were placed for refractory cardiogenic shock preoperatively in 6 patients, intraoperatively in 4 patients, and postoperatively as a rescue in 1 patient. Seven patients received Impella CP, 1 Impella RP, 1 Impella CP and RP, and 2 Impella 5.0. Additionally, 3 patients required preoperative venovenous extracorporeal membrane oxygenation (VV-ECMO), and 1 patient required intraoperative venoarterial extracorporeal membrane oxygenation (VA-ECMO). All Impella devices were removed 1 to 28 days after implantation. Length of stay in the intensive care unit stay ranged from 2 to 53 days (average 23.9±14.6). The 30-day and 1-year mortality were 0%. Ten of 11 patients were alive at 2 years. Also, 1 patient died 18 months after surgery from complications of coronavirus disease (Covid-19). Device-related complications included varying degrees> of hemolysis in 8 patients (73%) and device malfunction in 1 patient (9%).

**Conclusions:**

The Impella ventricular support system can be combined with other mechanical support devices for additional hemodynamic support. All patients demonstrated myocardial recovery with no deaths in the perioperative period and in 1-year of follow-up. Larger studies are necessary to validate these findings.

## INTRODUCTION

Cardiogenic shock is characterized by inadequate tissue perfusion related to cardiac dysfunction. Acute cardiogenic shock prior to or immediately after cardiac surgery is associated with high mortality rates, and thus hemodynamic support is required to ensure adequate end-organ perfusion^[1]^. Mechanical circulatory support devices have been shown to improve outcomes in patients with refractory cardiogenic shock when compared to the use of pharmacological agents or intra-aortic balloon pumps (IABPs)^[2]^. Recently, the use of short-term ventricular assist devices (VADs) has become a widely accepted treatment option for refractory cardiogenic shock. While pulsatile flow support is accomplished with an IABP, more powerful continuous flow is achieved with VADs like the Impella (Abiomed, Inc., Danvers, MA, USA) ventricular support system^[1]^. The purpose of this study is to report our single-surgeon experience on the role of Impella devices in patients with acute cardiogenic shock in the perioperative period of cardiac surgery at a private community hospital.

## METHODS

### Study Design

This is a retrospective review of a single-surgeon (KAP) and a single-center series of 11 consecutive patients who underwent placement of Impella devices from January 1, 2016 through October 1, 2019 in the perioperative period of cardiac surgery for refractory cardiogenic shock. Permission for studying these patients was obtained from our Institutional Review Board (F/N-R20-3930L) on December 4, 2019. Informed consent was waived due to the retrospective nature of the study. Patients were identified from a prospectively maintained database containing demographic, clinical, operative, and follow-up data. Mortality was assessed using the hospital’s database repository.

### Impella Ventricular Support System

The Impella ventricular support system is a family of temporary mechanical circulatory support devices consisting of a catheter-mounted microaxial flow pump that can be inserted percutaneously or directly across the aortic valve into the left ventricle (LV). It is designed to directly unload the LV, thereby reducing myocardial wall stress and oxygen consumption while increasing cardiac output and coronary and end-organ perfusion^[2,3]^.

**Fig. 1:**
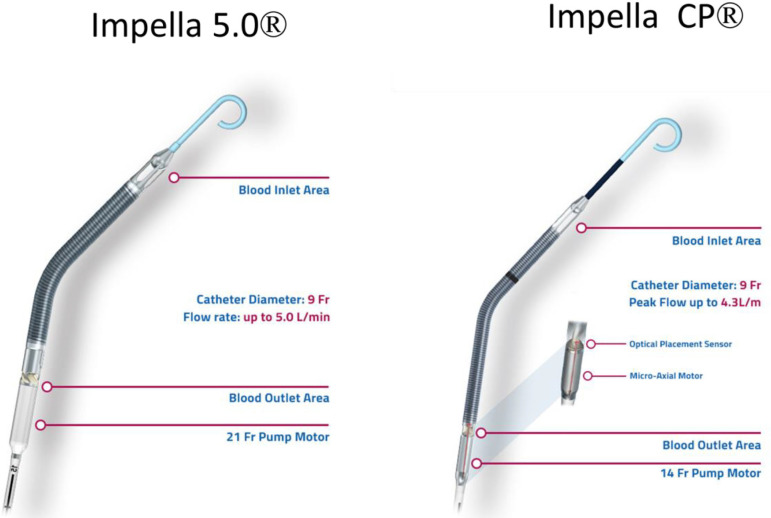
Impella 5.0 and Impella CP. Reprinted with permission from Abiomed, Inc. (Danvers, Massachusetts, USA), the manufacturer of the device.

**Fig. 2:**
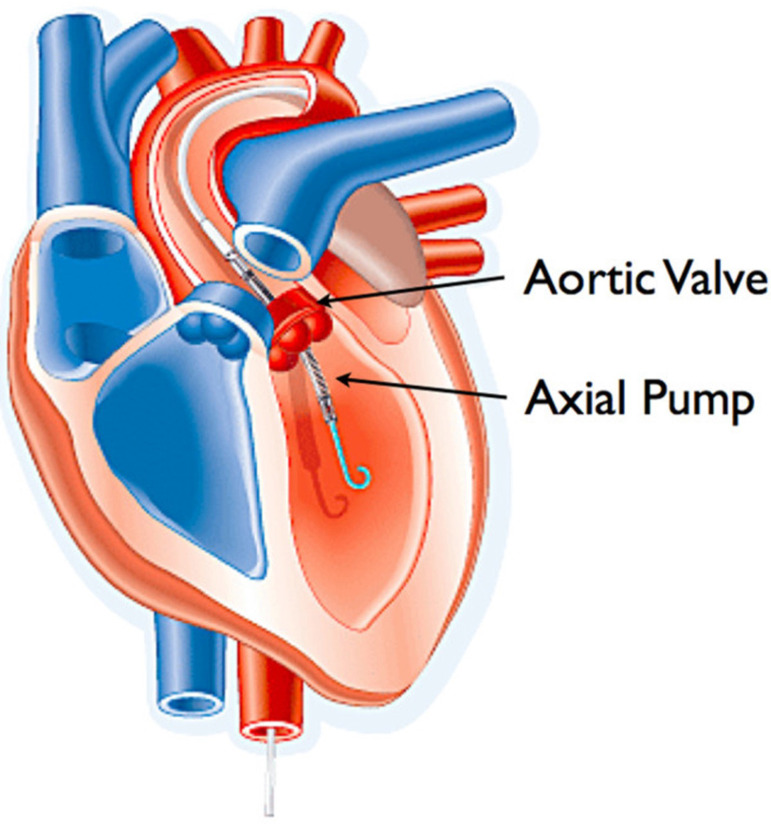
Diagram demonstrating the Impella 2.5 axial flow left ventricular assist device sitting across the aortic valve. Reprinted with permission from Abiomed, Inc. (Danvers, Massachusetts, USA.) the manufacturer of this device.

These devices have flexible pigtail-shaped tips followed by a cannula that contains the pump outlet and inlet areas, motor housing, and pump pressure monitor (Figures 1 and 2). The Impella 5.0® device is mounted on a 9 French (Fr) catheter shaft, and the pump is 21 Fr in diameter. It is inserted from transthoracic or transsternal access through a 10-mm vascular graft sewn end-to-side on the ascending aorta and advanced across the aortic valve into the LV. Alternatively, the device can be inserted peripherally from the femoral artery and advanced retrograde with transesophageal echocardiography guidance across the aortic valve into the LV. Impella 5.0 can generate flows up to 5.0 liters per minute. The Impella CP® has a pump diameter of 14 Fr, generates flows up to 4 liters per minute, and can be placed across the aortic valve within minutes with sheath-based direct arterial puncture. The Impella RP® is a right ventricular circulatory support platform that typically produces flows of approximately 4.0-4.5 liters/minutes. Like the left-sided Impella devices, the Impella RP has a flexible pigtail-shaped tip followed by a cannula that contains the pump outlet and inlet areas, motor housing, and pump pressure monitor. In addition, the RP has a three-dimensional shape to help guide placement into the main pulmonary artery.

### Impella Weaning Protocol

After removal of inotropes and vasopressors and when stable vital signs were present, the weaning protocol of Impella support consisted of monitoring hemodynamic and laboratory values (i.e., urine output, lactate, mixed venous, thermodilution calculation) to establish condition of end-organ perfusion. Once the parameters were stable, weaning was initiated by decreasing the pump performance in decrements of 2 levels and then assessing the patient for 1 hour. Once the performance level of the device was reduced to P2 level and recovery of LV function was established by transesophageal echocardiogram, the device was removed in the operating room.

### Statistical Analysis

Categorical data are presented as frequency (percentage). Continuous data are presented as mean+standard deviation (SD) or mean (range). Differences in ejection fraction before *versus* after Impella were normally distributed, and the average difference was analyzed using a two-sided paired t-test. Normality was assessed using a histogram and boxplot. Significance was assessed at the 0.05 level, and data was analyzed in Stata/MP 15.1 (StataCorp LP., Texas, USA).

## RESULTS

Eleven consecutive patients underwent placement of Impella devices: 6 preoperatively, 4 intraoperatively, and 1 postoperatively for refractory cardiogenic shock. Patient characteristics and demographics are reported in Table 1. Patients had multiple comorbidities, including coronary artery disease in 4 (36%) patients, congestive heart failure in 2 (18%) patients, mitral regurgitation (MR) in 7 patients (64%), aortic insufficiency (AI) in 3 patients (27%), and aortic stenosis (AS) in 2 patients (18%). Perioperative ejection fraction (EF) in all patients was 35.5±24.4%. Surgeries performed included coronary artery bypass graft (CABG) in 2 patients (18%), CABG and mitral valve replacement/repair in 3 patients (27%), aortic valve replacement/repair in 2 patients (18%), mitral valve replacement in 2 patients (18%), aortic and mitral valve replacement in 1 patient (9%), and emergent pulmonary embolectomy in 1 patient (9%).

**Table 1:** Patient demographics and clinical characteristics.

Variable	Impella preop N=6	Impella intraop N=4	Impella postop N=1	Impella - all N=11
Mean age (years)±SD	58.2±13.1	55.5±17.9	76 (N/A)	58.8±14.7
Male	4 (67%)	3 (75%)	0 (0%)	7 (64%)
Female	2 (33%)	1 (25%)	1 (100%)	4 (36%)
Coronary artery disease	1 (17%)	3 (75%)	0 (0%)	4 (36%)
Prior cardiac surgery	2 (33%)	0 (0%)	0 (0%)	2 (18%)
Congestive heart failure	2 (33%)	0 (0%)	0 (0%)	2 (18%)
Hypertension	3 (50%)	2 (50%)	0 (0%)	5 (45%)
Diabetes mellitus	2 (33%)	2 (50%)	0 (0%)	4 (36%)
COPD	2 (33%)	1 (25%)	0 (0%)	3 (27%)
Mitral regurgitation	4 (67%)	2 (50%)	1 (100%)	7 (64%)
Aortic insufficiency	2 (33%)	1 (25%)	0 (0%)	3 (27%)
Aortic stenosis	0 (0%)	1 (25%)	1 (100%)	2 (18%)
Hyperlipidemia	3 (50%)	3 (75%)	1 (100%)	7 (64%)
Chronic kidney disease	1 (17%)	1 (25%)	0 (0%)	2 (18%)

N/A=only 1 patient, standard deviation cannot be calculated. COPD=chronic obstructive pulmonary disease; SD=standard deviation

The indication for Impella insertion was refractory acute cardiogenic shock in all patients. The etiologies of the cardiogenic shock included severe coronary artery disease (CAD)±acute myocardial infarction (MI) in 5 patients (45%), valvular disease including severe MR±CAD in 7 patients (64%), severe AI in 1 patient (9%), severe AS in 2 patients (18%), and aortic valve endocarditis leading to severe AI and MR in 1 patient (9%) (Table 2). Two patients (18%) had an IABP placed in addition to Impella devices.

**Table 2:** Indications of Impella device.

Perioperative period	Patient#	Etiologies of cardiogenic shock	Device	Access	Duration of support (days)	ICU length of stay (days)	ECMO utilization
**Preoperative**	1	Acute MI led to papillary muscle rupture	CP	Groin	2	16	VV-ECMO (1 day)
	2	Unsuccessful septal myectomy	CP	Groin	2	2	VV-ECMO (2 days)
	3	MV dehiscence, CHF	CP	Groin	1	10	VV-ECMO (2 days)
	4	Acute *cor pulmonale* with thromboembolic disease led to RV dysfunction	RP	Groin	21	43	No
	5	Severe AS, severe MR	CP	Groin	4	33	No
	6	Acute right coronary occlusion following atherectomy	CP	Groin	<1	25	No
**Intraoperative**	1	Severe CAD, severe MR. Severe acute on chronic biventricular HF refractory to IABP	CP and RP	Groin	8-CP 6-RP Total=10	18	No
	2	Preoperative severe AS, bicuspid valve. Severe reduction in ventricular function (EF 10%), CHF, AVR	5.0	Axillary	19	26	No
	3	Multivessel coronary disease, recent MI, and severe MR. CABG, MV repair	5.0	Groin	28	53	VA-ECMO (13 days)
	4	Multivessel coronary disease, recent MI, ischemic cardiomyopathy (EF=15%), CABG	CP	Axillary	3	16	No
**Postoperative**	1	Severe AS and MR. Severe LV dysfunction, AVR, MV repair	CP	Groin	3	21	No

AS=aortic stenosis; AVR=aortic valve regurgitation; CABG=coronary artery bypass graft; CAD=coronary artery disease; CHF=congestive heart failure; ECMO=extracorporeal membrane oxygenation; EF=ejection fraction; HF=heart failure; IABP=intra-aortic balloon pump; ICU=intensive care unit; LV=left ventricle; MI=myocardial infarction; MR=mitral regurgitation; MV=mitral valve; RV=right ventricle; VA-ECMO=venoarterial extracorporeal membrane oxygenation; VV-ECMO=venovenous extracorporeal membrane oxygenation

Patient 6 in the preoperative Impella group had an IABP placed after Impella insertion in the cardiac catheterization suite due to a failed attempt at percutaneous coronary intervention (PCI) using a rotational atherectomy device that became lodged in the right coronary artery (RCA). The patient was emergently taken to the operating room for removal of the device and RCA bypass. Patient 4 in the intraoperative Impella group had an IABP placed temporarily during CABG due to low preoperative EF (10%) and need for significant inotropic support. The IABP was removed during surgery after successful insertion of the Impella CP device.

Three patients (36%) were placed on venovenous extracorporeal membrane oxygenation (VV-ECMO) in the preoperative group, and 1 patient (9%) was placed on venoarterial extracorporeal membrane oxygenation (VA-ECMO) with RP and Impella CP support in the intraoperative group (Table 2). Patient 3 in the intraoperative group required VA-ECMO placed intraoperatively in combination with the Impella CP device due to severe LV dysfunction and hemodynamic instability during CABG×5, mitral valve repair, and left ventriculotomy for resection of a left ventricular thrombus. VV-ECMO, used in 3 patients of the preoperative group for 1-2 days (Table 2), was removed before Impella support in 2 patients and after Impella in 1 patient. VA-ECMO, used in the patient of the intraoperative group for 13 days (Table 2), was removed 15 days before the removal of the Impella support.

Access sites for Impella device insertion included femoral artery in 9 patients (82%) and axillary artery in 2 patients (18%). The access site for the Impella RP was the right femoral vein. The devices utilized in this study were Impella CP in 7 patients, Impella 5.0 for LV support in 2 patients, Impella RP for right ventricular support in 1 patient, and Impella CP and RP in 1 patient. Patients required Impella support for an average of 8.5 days (range <1 to 28) for all 3 perioperative groups. Intensive care unit stay ranged from 2 to 53 days (average of 23.9±14.6 days) (Table 2).

Myocardial recovery was demonstrated in all patients. Mean EF for all patients increased significantly from 35.5%+24.4% before Impella to 46.8+20.0% after Impella (*P*=0.037). Device-related complications included varying degrees> of hemolysis in 8 patients (73%) and device malfunction in 1 patient (9%). Patient 1 (intraoperative) with the device malfunction had both RP and Impella CP support as well as VA-ECMO. While attempting a chest closure, the patient developed right ventricle (RV) dysfunction: a Protek Duo right ventricular assist device (RVAD) was placed percutaneously.

Other surgical-related complications reported in Table 3 included pneumonia in 5 patients (45%), ventilator-dependent respiratory failure (VDRF) requiring tracheostomy in 4 patients (36%), paroxysmal atrial fibrillation (AF) in 3 patients (27%), ventricular arrhythmias requiring placement of automatic implantable cardioverter defibrillator (AICD) in 2 patients (18%), heparin-induced thrombocytopenia in 2 patients (18%), superficial wound infection in 1 patient (9%), and limb ischemia requiring fasciotomy in 1 patient (9%). The 30-day and 1-year mortality were 0%. Also, 10 of 11 patients were alive at 2 years. One patient (preoperative patient #2) died 18 months after surgery from complications of coronavirus disease (Covid-19).

**Table 3:** Device-related and patient complications.

Device-related complications	N (%)
Hemolysis	8 (73%)
Device malfunction	1 (9%)
	
**Patient complications**	
Pneumonia	5 (45%)
VDRF requiring tracheostomy	4 (36%)
Paroxysmal atrial fibrillation	3 (27%)
Ventricular arrhythmias requiring AICD	2 (18%)
Sternal wound infection	1 (9%)
Limb ischemia requiring fasciotomy	1 (9%)
Heparin-induced thrombocytopenia	2 (18%)
30-day mortality	0 (0%)
1-year mortality	0 (0%)

AICD=automatic implantable cardioverter-defibrillator; VDRF=ventilator-dependent respiratory failure

## DISCUSSION

Despite improvements in therapeutic options available in recent years, cardiogenic shock is associated with mortality rates exceeding 50%^[4]^. Therapeutic options available include inotropic pharmacological therapy with or without IABP, VA-ECMO, and more recently, short-term acute mechanical devices like Impella^[1,4,5]^. The superior hemodynamic effects of the Impella ventricular support system, mainly related to its unloading LV capacity, as well as its relative ease of insertion, have led to its preferred use to mitigate the deleterious outcomes and high mortality rate associated with cardiogenic shock. Based on favorable outcome data from the RECOVER I trial (multicenter prospective study of Impella 5.0/LD for postcardiotomy circulatory support conducted between October 2006 and May 2008) and the USpella Registry (154 patients with acute MI complicated by cardiogenic shock (AMICS), treated with combined PCI and Impella between June 2009 and March 2012), the Food and Drug Administration granted approval in the United States of Impella 2.5 in 2008 and Impella CP in 2012^[6,7]^. More than 50,000 Impella devices have been implanted to date in the U.S. at more than 1,000 sites^[3]^.

The present study includes a consecutive series of 11 patients undergoing placement of Impella devices for refractory acute cardiogenic shock in the perioperative period of cardiac surgery. Impella devices were placed preoperatively in 6 patients, intraoperatively in 4 patients, and postoperatively in 1 patient. Indication for Impella insertion was acute cardiogenic shock of diverse etiologies including severe CAD±MI, severe MR±CAD, severe aortic valve regurgitation (AVR) and severe AS, and aortic valve endocarditis leading to severe AVR and MR. In 1 patient, Impella RP was used with Impella CP for biventricular heart failure. In another patient, Impella RP was used for thromboembolic disease leading to RV dysfunction. In 5 patients, VV-ECMO was used together with Impella. In 1 patient, VA-ECMO was used with Impella. In 1 patient, an IABP was placed temporarily after Impella insertion in the cardiac catheterization suite due to a failed PCI attempt. In another patient, IABP was temporarily placed intraoperatively during CABG surgery for ischemic cardiomyopathy due to a very low EF and need for significant inotropic support. IABP was removed during surgery after successful insertion of the Impella CP device. Similarly, in other studies, indications for using Impella were acute MI complicated by cardiogenic shock, to facilitate high-risk PCI, cardiomyopathy with acute decompensation, postcardiotomy cardiogenic shock, and off-pump coronary artery bypass surgery^[2]^.

Survival rate at 30 days and 1 year was 100%, with no deaths in the perioperative period and in the 1-year follow-up. This compares favorably with other studies. Lemaire et al.^[1]^, in a retrospective study of 47 patients who underwent placement of Impella for cardiogenic shock, observed a survival rate at 30 days, 90 days, and 1 year of 72.3%, 65.9% and 63.8%, respectively. In a meta-analysis from 6 studies in which Impella devices (2.5 or 5.0) were used for cardiogenic shock in 10 or more patients (excluding patients with concomitant IABP), Batsides et al.^[4]^ found a pooled rate of survival at 30, 180, and 365 days of 72.6%, 62.7%, and 58.4%, respectively. In 40 patients requiring Impella support for more than 6 hours, Badiye et al.^[8]^ observed a 30- and 90-day survival of 65% and 60%, respectively. Myocardial recovery was observed in all 11 patients in this study. Lemaire et al.^[1]^ observed a myocardial recovery of 72% (34 of 47 patients), whereas Batsides et al.^[4]^ found a recovery rate of 73.8% in a pool of 2 studies including 24 patients.

All patients in this study were successfully weaned off of all mechanical support devices. Average time of support of the Impella device was 8.5 days (range <1 to 28) days after placement. These results are comparable to other studies. Lemaire et al.^[1]^ observed a duration of Impella of 5.4 (1 to 18) days, with 64% of the patients having the device removed within that period, and 9% being transitioned to long-term support. Batsides et al., in a pool of 6 studies, described 8.6 days (1 to 71) prior to removal of Impella^[4]^. In Badiye et al.^[8]^, 40 patients with Impella were placed for more than 6 hours and showed an average time of support of 86.63 hours. den Uil et al.^[9]^, in 6 studies with Impella 2.5 support predominantly in patients with cardiogenic shock from acute MI, reported a mean support time of 1.6±2.7 days, and in 4 studies with Impella 5.0, a mean support time of 6.1±3.9 days.

The use of Impella devices preoperatively, in some cases in combination with other mechanical support devices, can prevent catastrophic events in patients with cardiogenic shock. Additionally, VV-ECMO can be particularly useful in preoperative stabilization after acute cardiac or respiratory failure in patients before cardiac surgery and refractory postcardiotomy hypoxia. The prophylactic use of Impella devices in cardiac surgery may lead to faster myocardial recovery and improved outcomes. Flaherty et al.^[10]^ observed that early implantation of Impella (placement either before revascularization or early on during angiography) in acute MI complicated by cardiogenic shock decreased in-hospital or 30-day mortality by 48% compared with late initiation of Impella (post-revascularization). Basir et al.^[11]^ showed that placement of Impella prior to the use of PCIs, inotropes, or vasopressors improved survival in patients with cardiogenic shock. Survival was 66% when Impella was initiated less than 1.25 hours from shock onset, 37% when initiated within 1.25 to 4.25 hours, and 26% when initiated after 4.25 hours^[11]^. A recent study from Sabra et al.^[12]^ demonstrated that postoperative use of Impella to support high-risk patients undergoing CABG allowed the procedure to be performed in patients with depressed EF, thus improving the postoperative course with results comparable to IABP.

Combining mechanical circulatory support with VA-ECMO and Impella is an attractive option for greater hemodynamic support. Prolonged use of VA-ECMO alone may lead to increased LV afterload as well as worsening AI. Concomitant use of Impella devices with VA-ECMO in these patients may limit these complications. Combined use of VA-ECMO and Impella has been shown to reduce mortality at 30 days and 1 year and to decrease the need for inotropic agents^[13]^. Although often used simultaneously with other devices (IABP, VA-ECMO), Impella devices have been shown to be superior for their capacity to directly unload the LV and reduce myocardial workload, playing a significant role in myocardial recovery^[1-4,14]^. Lemor et al.^[14]^, comparing Impella *versus* VA-ECMO outcomes in patients with acute MI with cardiogenic shock, observed that the use of Impella was associated with better clinical outcomes, fewer complications, shorter length of hospital stay, and lower hospital costs compared to patients undergoing VA-ECMO placement.

Hemolysis was the most common complication related to the Impella device and likely related to implant duration. Varying degrees> of hemolysis were observed in 8 patients (73%), limb ischemia requiring fasciotomy in 1 patient (9%), and device malfunction in 1 patient (9%). Other complications included pneumonia in 5 patients (45%), VDRF in 4 patients (36%), paroxysmal AF in 3 patients (27%), ventricular arrhythmias requiring AICD in 2 patients (18%), heparin-induced thrombocytopenia in 2 patients (18%), and sternal wound infection in 1 patient (9%). Lemaire et al.^[1]^ described complications following Impella in 14 patients (30%), including device malfunction, high purge pressures, tube fracture, and groin hematoma. Batsides et al.^[4]^, in a meta-analysis of 163 patients with Impella (6 studies), observed a complication rate of 0.1% stroke, 21.6% bleeding, 0.2% limb ischemia, 0.7% hemolysis, 10.7% device malfunction, and 0.2 % valve injury. Badiye et al.^[8]^ described an incidence of hemolysis of 62.5% in 46 patients in whom Impella was used for more than 6 hours for cardiogenic shock.

### Limitations

The limitations of this study are its small sample size and retrospective nature. The indications for Impella support were also diverse. Also, 6 of 11 patients (55%) had other forms of mechanical support combined with Impella for additional hemodynamic support.

## CONCLUSIONS

All patients demonstrated myocardial recovery with no deaths in the perioperative period and in the 1-year follow-up, demonstrating improved survival outcomes compared to previous reports. Successful Impella support in cardiogenic shock may be further enhanced in combination with other mechanical support devices. These results from a single-surgeon experience are intended to be hypothesis-generating in nature and to serve as a reference for future, well-powered prospective studies.
